# Meropenem plasma concentrations in critically ill patients treated with the novel multi organ replacement therapy ADVOS

**DOI:** 10.1007/s15010-025-02554-4

**Published:** 2025-05-21

**Authors:** David Totschnig, Theresa Mader, Thomas Stimpfl, Klaus Breinbauer, Cristina Groza, David Stücklschwaiger, Stephanie Neuhold, Emanuela Friese, Johannes Holbik, Martina Delivuk, Tom Ripplinger, Marcell Leber, Clemens Ott, Wolfgang Hoepler, Aritz Perez Ruiz de Garibay, Christoph Wenisch, Otto Frey, Alexander Zoufaly, Marianna Traugott

**Affiliations:** 1 Department of Medicine IV, Klinik Favoriten, Vienna Healthcare Group, Kundratstraße 3, 1100 Vienna, Austria; 2 Department of Laboratory Medicine, University Hospital Vienna, Währinger Gürtel 18-20, 1090 Vienna, Austria; 3 Apotheke Kliniken Landkreis Heidenheim gGmbH, Regionales Arzneimittelinformationszentrum, Schloßhaustraße 100, 89522 Heidenheim, Germany; 4 ADVITOS GmbH, Agnes-Pockels-Bogen 1, 80992 Munich, Germany; 5 Faculty of Medicine, Sigmund Freud Private University Vienna, Freudplatz 3, 1020 Vienna, Austria

**Keywords:** Meropenem, TDM, Therapeutic drug monitoring, ADVOS, Multi organ replacement therapy, Calculated vs. measured values

## Abstract

**Background:**

Optimal dosing of antibiotics in critically ill patients treated with the novel multi organ replacement therapy ADVOS (ADVanced Organ Support) based on albumin dialysis is unclear. This study aims to provide real life data on meropenem plasma concentrations after prolonged infusion in patients treated with ADVOS and a critically ill control group with and without continuous veno-venous hemodiafiltration (CVVHDF).

**Methods:**

We retrospectively analyzed plasma concentrations of meropenem obtained as part of our standard of care therapeutic drug monitoring in the intensive care unit. Meropenem was administered as a prolonged infusion over 3 h. We measured peak and trough levels, pre-and post-filter levels of meropenem using high performance liquid chromatography. We calculated the meropenem clearance and compared the measured clearance with predicted clearance based on creatinine, calculated by the MeroEasy tool.

**Results:**

In total, 159 measurements across 16 patients were analyzed. Meropenem trough concentrations were highest in the CVVHDF group with a median of 23.5 mg/L, followed by the ADVOS (median 9.3 mg/L) and control group (median 7.6 mg/L). No trough levels were below the lower limit of 2 mg/L in the CVVHDF and ADVOS groups. Meropenem machine clearance by CVVHDF was calculated to be 1.8 (± 0.5) L/h and 3.5 (± 1) L/h for ADVOS.

**Conclusion:**

Our results suggest that ADVOS treatment in critically ill patients receiving a high dose meropenem regimen (2 g IV q8h) does not lead to underdosing. Some trough values were even within potentially toxic levels, especially in the CVVHDF group, highlighting the importance of therapeutic drug monitoring.

**Supplementary Information:**

The online version contains supplementary material available at 10.1007/s15010-025-02554-4.

## Introduction

Infections are a leading cause of morbidity and mortality among critically ill patients in intensive care units (ICUs). The management of these infections requires timely and effective antibiotic therapy to combat potentially life-threatening pathogens. Beta-lactam antibiotics, particularly meropenem, play a crucial role due to their broad-spectrum activity and favorable safety profile [1]. However, ensuring optimal dosing in this patient population is a persistent challenge due to complex pharmacokinetic changes and variability in clinical conditions [2, 3].

Meropenem is widely used in ICUs to treat severe bacterial infections, including those caused by more resistant organisms [4]. Its pharmacokinetics are characterized by low protein binding and high renal clearance. As a hydrophilic molecule it has a low volume of distribution of 0.4–0.6 L/kg in critically ill patients, making it effective for bloodstream infections [3]. The efficacy of beta-lactam antibiotics depends on maintaining drug concentrations above the pathogen's minimum inhibitory concentration (fT > MIC) [5]. In ICU patients treated with intermittent meropenem infusions trough levels up to 2–4 times above the MIC are targeted. The MIC for meropenem sensible pathogens is ≤ 2 mg/L, hence meropenem trough levels of 4–8 mg/L are targeted in severely ill patients [2].Underdosing can result in treatment failure, while excessive meropenem concentrations risk neurotoxicity and nephrotoxicity [6]. In critically ill patients, pharmacokinetics are often altered by factors such as renal dysfunction, hyperfiltration, fluid overload, and the use of extracorporeal therapies, such as continuous veno-venous hemodiafiltration (CVVHDF) [2, 3] or the ADVanced Organ Support (ADVOS) system. The latter is an innovative extracorporeal therapy that integrates albumin dialysis with multi-organ support, effectively removing protein-bound substances like bilirubin and addressing acid–base disorders [7]. It is particularly valuable in patients with multi-organ failure, sepsis, or profound shock due to hepatic or renal dysfunction [8–10]. For extracorporeal therapies therapeutic drug monitoring (TDM) is recommended to optimize dosing [2].

The effects of novel systems such as ADVOS on antibiotic clearance remain unclear. While traditional systems like CVVHDF have been studied, limited data exists regarding the clearance of antibiotics like meropenem during ADVOS therapy [11, 12]. Dosing calculators, such as the *MeroEasy* tool (https://www.doseeasy.de/) and *the Caddy* (https://www.thecaddy.de/caddy/caddy/), are not validated for ADVOS, further complicating dose optimization in these patients.

This study aims to address the knowledge gap by investigating the pharmacokinetics of meropenem in critically ill patients undergoing ADVOS therapy. Specifically, it seeks to compare meropenem plasma levels in patients treated with ADVOS versus those on CVVHDF or without extracorporeal therapy. These real-world data will help optimize antibiotic treatment strategies and improve outcomes in this complex patient population.

## Methods

### Study design

This retrospective observational study was conducted in the intensive care unit (ICU) of a tertiary care hospital specialized on infectious diseases. It aimed to evaluate the pharmacokinetics of meropenem in critically ill patients, comparing plasma concentrations and clearance rates across the three patient groups. Plasma concentrations of meropenem, collected as part of standard therapeutic drug monitoring (TDM) between October 2022 and February 2024, were analyzed. Data were extracted from electronic medical records, including demographic and clinical characteristics, renal function, organ support modalities, and antibiotic dosing regimens.

All included patients were critically ill and required ICU treatment for several days. Patients were categorized into three groups based on the presence and type of extracorporeal therapy: (1) patients on ADVOS therapy (ADVITOS^®^, Munich, Germany), (2) patients on continuous veno-venous hemodiafiltration (CVVHDF; Prismaflex^®^, Baxter^®^, Deerfield, USA), and (3) patients without extracorporeal organ replacement therapy.

### Interventions

All patients received meropenem via prolonged infusion over three hours using a perfusion pump. After an initial bolus of 2 g, subsequent dosing was determined according to the European Committee on Antimicrobial Susceptibility Testing (EUCAST) high-dose recommendations based on glomerular filtration rate (GFR): 1) GFR > 50 mL/min/1.73m^2^: 2 g every 8 h; 2) GFR 30–50 mL/min/1.73m^2^: 2 g every 12 h; 3) GFR < 30 mL/min/1.73m^2^: 1 g every 12 h (control group without dialysis) [20].

For patients on ADVOS or CVVHDF, the maximum approved dose of 2 g every 8 h was used with the aim to achieve sufficient plasma levels to cover pathogens with a MIC up to 4 mg/L (i.e., Epidemiological cut-off value—ECOFF for Acinetobacter) [20].

### Technical settings of the extracorporeal therapies

ADVOS: A blood flow of 100 mL/min and a concentrate flow of 160 mL/min were used in all patients.

CVVHDF: A blood flow of 100 mL/min was used in all patients. The substitute was re-infused as post-dilution. In most patients the dialysate flow and substitute flow were both 750 mL/min. Prismaflex ST150 filters (Baxter®, Deerfield, USA) were used.

Citrate was used as anticoagulation method in CVVHDF and ADVOS.

### Sampling

Our meropenem treatment was always started with an initial bolus of 2 g i.v. which was followed by a prolonged infusion of 2 g over 3 h. Plasma samples were taken at the earliest after the first prolonged infusion (peak value) and already on the first day of treatment with the extracorporeal device. Samples were drawn at two time points per dosing interval: 1) Peak levels: 1–20 min after the end of the infusion; and 2) trough levels: 1–20 min before the next scheduled dose. All patients were sampled during multiple dosing intervals.

In patients receiving ADVOS or CVVHDF, both pre- and post-filter samples were obtained simultaneously with maximum 2 min time lag. Samples were only taken if the patient was receiving the extracorporeal treatment during the entire duration of the dosing interval (time between start of the prolonged infusion and trough measurement before the next prolonged infusion). Reasons for interruptions of the extracorporeal treatment were e.g. planned changes of the hose set (CVVHDF), planned changes of the machine (ADVOS) or unplanned problems with the blood flow or problems with the extracorporeal devices. Blood was drawn into EDTA vials via arterial access, immediately centrifuged (10 min at 3,000 × g), and plasma aliquoted into vials containing 50 µL stabilizing priming solution (Chromsystems^®^, Munich, Germany). Samples were mixed, frozen at −20 °C, and transported under controlled conditions to the University Hospital Vienna for analysis the next workday. Samples arriving uncooled were discarded.

Meropenem plasma concentrations were measured using high-performance liquid chromatography (HPLC) with the CE-IVD-certified reagent kit "Antibiotics in serum/plasma" (Identification number: 61000, Chromsystems^®^, Munich, Germany).

### Clearance calculations

Meropenem clearance attributed to ADVOS (CL_ADVOS_) and to CVVHDF (CL_CVVHDF_) were calculated using pre- and post-dialyzer meropenem levels and blood flow set during treatments, as stated in Eq. 1.

Equation 1. CL_ADVOS_ and CL_CVVHDF_ were calculated considering meropenem levels pre- and post-dialyzer and the blood flow (L/h) set during treatments.

$$CL=\frac{{Meropenem}_{pre}-{Meropenem}_{post}}{{Meropenem}_{pre}}\times Blood Flow (L/h)$$

Overall clearances based on measured meropenem trough levels (CL_patient_) and estimated clearances based on creatinine values (CL_estimated_) were calculated using the *MeroEasy* tool (https://doseeasy.de/). The calculator considers sex, age, body weight, height and serum creatinine to estimate meropenem levels.

Detailed information and formulas regarding the MeroEasy tool can be found in supplementary information.

### Statistical analysis

Descriptive statistics were used to summarize patient characteristics and measured values. Meropenem trough concentrations were compared between groups using the Mann–Whitney test, while clearance values were compared using paired t-tests where applicable. Graphical representations and statistical analyses were performed using GraphPad Prism 9 (Dotmatics, Boston, MA, USA). A p-value < 0.05 was considered statistically significant.

## Results

The patient characteristics are detailed in Table 1. We included 16 patients (11 male/5 female) in our analysis. Two patients had measurements in both the ADVOS and CVVHDF group, resulting in a total of 8 patients in the control group without dialysis, 5 in the CVVHDF group and 5 in the ADVOS group. Creatinine at baseline was lower in the control group with a median of 0.6 mg/dL vs. 1.4 mg/dL and 1.3 mg/dL in CVVHDF and ADVOS patients respectively. Bilirubin at baseline was highest in the ADVOS group with a median of 3.9 mg/dL. All patients groups were critically ill with mean APACHE II score > 20 [13]. Patients in the CVVHDF and ADVOS group were most ill with a median APACHE II score of 30 and 29, respectively. No statistically significant differences were found between groups for any of the baseline and demographic parameters. As signs of potential neurotoxicity, delirium was documented in 10 out of 16 patients, and seizures occurred in 2 out of 16 patients, both with underlying neurological disorders.

**Table 1:** Patient characteristics. Median (IQR25, IQR75). P-values from one-way ANOVA testing for differences between groups for each clinical variable. Pairwise comparisons did not reveal significant differences either (data not shown)

	Control	CVVHDF	ADVOS	P
Number of patients	8	5	5	
Number of measurements	81	33	45	
Gender (Male/Female)	6/2	4/1	2/3	
Age (years)	63 (47, 74)	62 (59, 63)	60 (58, 61)	0.880
Weight (kg)	74 (63, 84)	95 (90, 96)	75 (65, 97)	0.680
Height (cm)	170 (166, 175)	170 (170, 180)	170 (168, 170)	0.353
Creatinine at baseline (mg/dL)	0.6 (0.5, 1.0)	1.4 (1.0, 1.7)	1.3 (1.3, 1.4)	0.713
GFR at baseline (CKD-EPI, ml/min/1.7m^2^)	90 (58, 90)	48 (42, 84)	45 (43, 71)	0.405
Bilirubin at baseline (mg/dL)	0.5 (0.4, 1.2)	2.8 (0.8, 6.1)	3.9 (2.8, 13.9)	0.090
Albumin at baseline (g/L)	22.1 (21.6, 23.1)	23.6 (22.2, 23.6)	23.6, 23.3, 27.1)	0.795
APACHE II Score	21.5 ( (8.5, 28.5)	30 (17.5, 35.5)	29 (17.5, 41)	0.321

**Graph 1:**
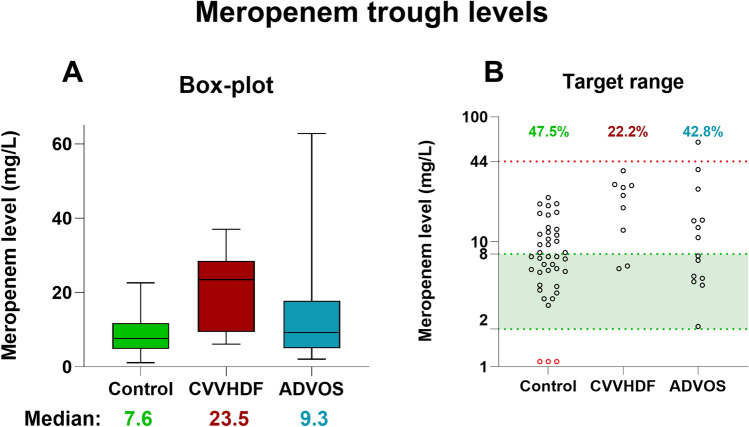
Analysis of meropenem trough levels, **A** Min to max Boxplot of trough levels in the control, CVVHDF- and ADVOS group. **B** Scatterplot of all trough levels. The green area highlights the target range of trough levels of 2 – 8 mg/L (20), the red line shows nephrotoxic levels of 44 mg/L (6). The percentages at the top show the fraction of values in the target range area. *CVVHDF* continuous veno-venous hemodiafiltration, *ADVOS* ADVanced Organ Support

Meropenem trough levels in the CVVHDF group (median 23.5 mg/L, IQR: 12.4, 28.2) were higher than those in the ADVOS (median 9.3 mg/L, IQR: 5.2, 14.9) and control groups (median 7.6 mg/L, IQR: 5.9, 11.9) (Graph 1A). The difference was only significant between the CVVHDF and control groups (p = 0.0017), but not between CVVHDF and ADVOS (p = 0.12). Trough levels in the ADVOS group varied strongly from 2.1 to 62.8 mg/L. In the control group 47.5% of trough levels were within the optimal range – with 3 levels below the optimal range (Graph 1B). Similarly, 42.8% of the patients in the ADVOS group were within the target zone. In the CVVHDF group only 22.2% were within the target zone.

**Graph 2:**
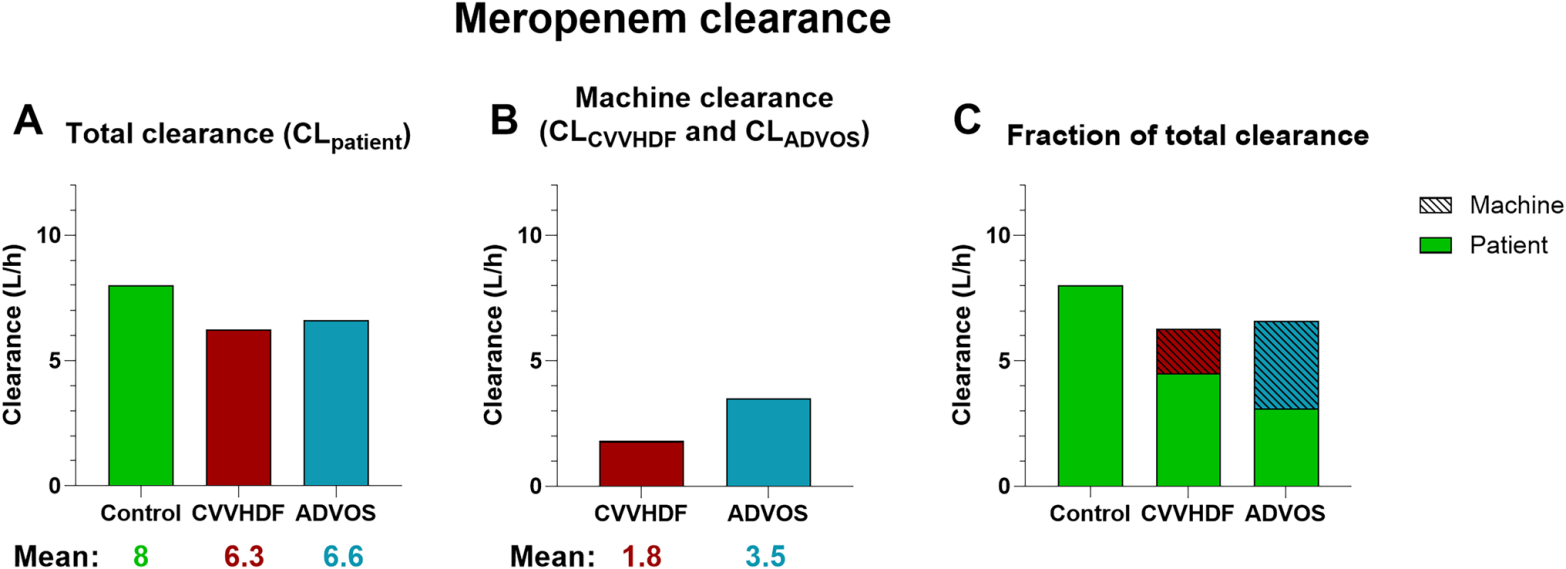
Analysis of meropenem clearance, **A** Bar graph of calculated meropenem clearance (CL_patient_) based on trough levels. **B** Bar graph of calculated machine clearance (CL_ADVOS_ and CL_CVVHDF_), based on measured pre- and post-dialyzer levels. **C** Bar graph showing the fraction of total clearance achieved by the respective machine clearance. Striped areas represent the machine clearance. Estimated values by subtracting the machine clearance (CL_ADVOS_ and CL_CVVHDF_), (**B**) from the total clearance (CL_patient_) (**A**). *CVVHDF* continuous veno-venous hemodiafiltration; *ADVOS* ADVanced Organ Support

Next, we analyzed the meropenem clearance in our patients (Graph 2). The meropenem clearance calculated based on the trough values (CL_patient_) was highest in the control group with a mean of 8 L/h (± 2), followed by the ADVOS (6.6 ± 2.9) and CVVHDF (6.3 ± 2.7) group (Graph 2A). The median (IQR25, 75) values were 7.9 (6.3, 9.3), 6.4 (4.8, 8.4) and 5.3 (4.4, 7.8) for the control, ADVOS and CVVHDF groups respectively. Furthermore, we calculated the machine clearance by using pre- and post-dialyzer values (Table 2) and found a mean clearance of 1.8 L/h (± 0.5) in the CVVHDF group (CL_CVVHDF_) and 3.5 L/h (± 1) in the ADVOS group (CL_ADVOS_) (Graph 2B). The median (IQR25, 75) values were 2.0 (1.3, 2.2) and 4.0 (3.0, 4.3) respectively. Graph 2 C shows a combination of the previous graphs, highlighting the fraction of total clearance achieved by the machine.

**Table 2:** Measured meropenem plasma-levels (mg/L). Median (IQR25, IQR75). Post-dialyzer values are not applicable (= n. a.) in the control group

Meropenem levels (mg/L)	Control (n = 81)	CVVHDF (n = 33)	ADVOS (n = 45)
*Peak levels*			
Pre-dialyzer	45.0 (35, 52)	43.3 (30.1, 51.9)	37.9 (20.4, 45.4)
Post-dialyzer	n.a	25.5 (23.4, 37.6)	11.0 (6.5, 13.0)
*Trough levels*			
Pre-dialyzer	7.6 (5.9, 11.9)	23.5 (12.4, 28.2)	9.3 (5.2, 14.9)
Post-dialyzer	n.a	14.9 (7.3, 19.6)	3.5 (3.3, 4.1)

**Graph 3:**
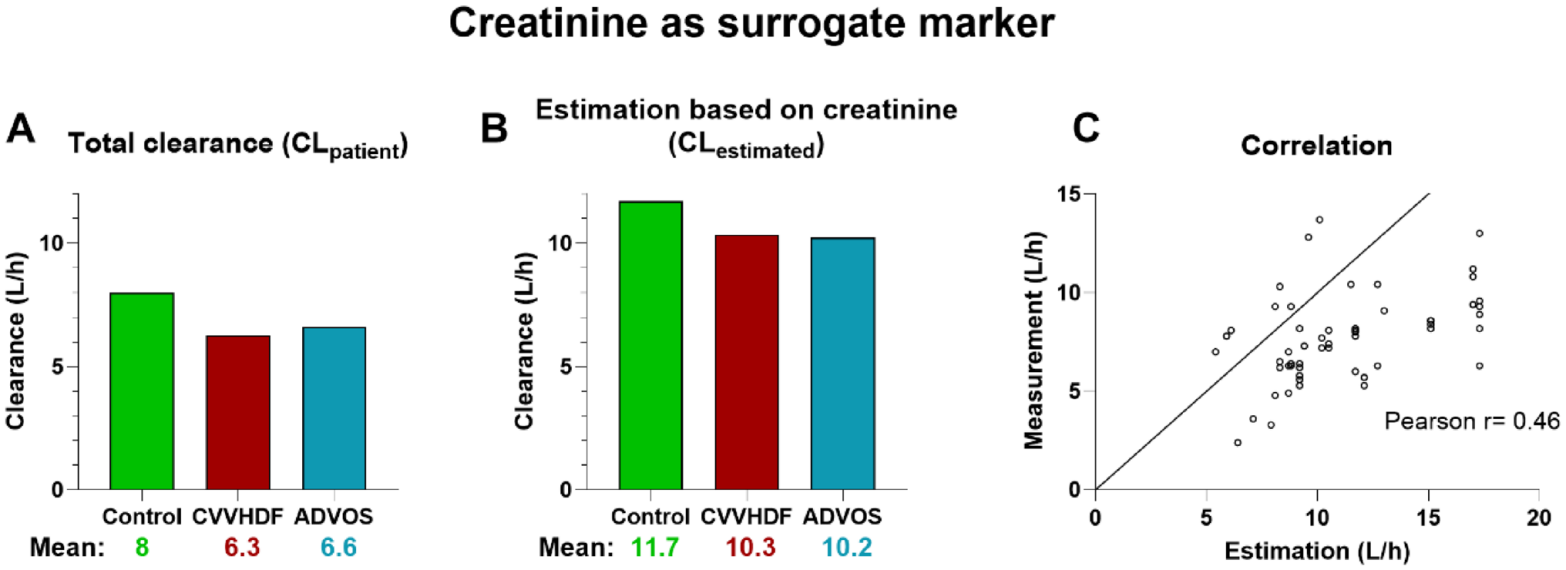
Analysis of creatinine as a surrogate marker, **A** Bar graph of calculated meropenem clearance, based on trough levels (CL_patient_). **B** Bar graph of estimated meropenem clearance, based on creatinine values (CL_estimated_). **C** Correlation plot of the measured meropenem clearance (**A**) and estimated meropenem clearance (**B**). *CVVHDF* continuous veno-venous hemodiafiltration, *ADVOS* ADVanced Organ Support

Finally, we examined whether estimated clearance values using creatinine (CL_estimated_) could be used as a surrogate marker for meropenem levels, potentially foregoing therapeutic drug monitoring. Graph 3B shows estimated meropenem clearance values (CL_estimated_) calculated with the *MeroEasy* tool based on the creatinine values measured in routine laboratory examinations on the day of the corresponding meropenem measurement. The estimated clearance based on creatinine levels was significantly higher than the estimated clearance based on measured plasma trough values (CL_patient_), with a mean difference of 3.7 L/h (p = 0.0001) for the control group, 4.1 L/h (p = 0.003) for the CVVHDF group and 3.6 L/h (p = 0.0003) for the ADVOS group. Correlation between the estimated and measured values was weak, with a pearson r of 0.46 (p = 0.0004) (Graph 3C).

## Discussion

This study provides the first real-life pharmacokinetic analysis of meropenem in critically ill patients undergoing ADVOS therapy and compares it with CVVHDF and non-extracorporeal treatments. It is also the first study to measure pre- and post-filter levels of meropenem to calculate machine-specific clearance in these patient groups.

The key findings indicate that ADVOS therapy is safe for critically ill patients treated with meropenem, as meropenem clearance by ADVOS (CL_ADVOS_) was lower than anticipated, and no trough levels fell below the therapeutic target of 2–8 mg/L. The ADVOS machine clearance (CL_ADVOS_) of 3.5 L/h, measured in this study, is comparable to the documented in vitro values (3.4 L/h) [12]. This is only approximately 30% of the normal intrinsic meropenem clearance in healthy adults (12.5 L/h [14]) or 40% of the clearance observed by Jaruratanasirikul et al. in non-renal insufficient intensive care patients (7.8 L/h) [15]. This clearance in critically ill patients is consistent to the clearance of 8 L/h measured in our control group (CL_patient_). Importantly, only 3 trough levels below 2 mg/L were observed, all in the control group without extracorporeal therapy. However, a significant number of trough levels exceeded the optimal range of 2–8 mg/L, particularly in the CVVHDF group (77.8%), compared to the ADVOS (57.2%) and control groups (52.5%).

These results align with previous in vitro studies and case reports on ADVOS König et al. reported meropenem ADVOS clearances between 3.4 and 6.08 L/h in an in vitro model [12], depending on flow rates. Similarly, she described a dialyzer clearance of 6.3 L/h in a patient treated with ADVOS at a higher blood flow rate (250 mL/min). Both findings support our observed ADVOS clearance of 3.5 L/h at lower flow rates (100 mL/min)​​. All our patients were treated with 100 mL/min blood flow to ensure hemodynamic stability and to avoid disturbances of the blood flow. Higher blood flow rates to treat severe acidosis more effectively were not necessary in our patient cohort.

These findings demonstrate that the limited ADVOS machine clearance of 3.5 L/h at a blood flow of 100 mL/min cannot compensate for the complete loss of renal function, with an assumed renal meropenem clearance in ICU patients of approximately 8 L/h. Hence, meropenem underdosing in patients with renal failure on ADVOS therapy receiving a high dose meropenem regimen (2 g q8h) is unlikely with these flows. Nonetheless, the ADVOS clearance is much more effective than the CVVHDF clearance (3.5 vs 1.8 L/h) and this must be considered in dosing. In CVVHDF, higher trough levels are consistent with existing studies that show impaired intrinsic clearance in patients with renal failure, even with extracorporeal therapy [16]. Prior studies on CVVHDF also highlight the unpredictability of antibiotic clearance due to variability in flow rates, membrane permeability, and patient-specific factors [16, 17]. The findings from this study further underscore the necessity of therapeutic drug monitoring (TDM) in CVVHDF to avoid overdosing, as the use of tools like *MeroEasy*, which relies on population estimates, may overestimate clearance without accounting for real-world machine maintenance pauses or individual variations. We recommend using the *MeroEasy* tool, when using continuous renal replacement therapies (CRRT, ADVOS), to adjust the dosage based on measured trough levels. Similarly, a recent study investigating hemoadsorption with CytoSorb® coupled post-filter to continuous renal replacement therapy reported no significant impact of this device in meropenem or piperacillin levels [18], emphasizing that the impact of extracorporeal therapies on antibiotic clearance is highly modality-specific and must be evaluated on a case-by-case basis.

In our study, the moderate correlation between estimated and measured meropenem clearance (r = 0.46, Graph 3C) highlights the limitations of creatinine-based estimation tools like *MeroEasy* in critically ill patients, particularly those undergoing extracorporeal therapies. These tools rely on stable renal function and demographic parameters, but do not account for the clearance contributed by ADVOS or CVVHDF. Moreover, serum creatinine often poorly reflects acute changes in kidney function and is affected by fluid shifts, inflammation, and muscle catabolism common in ICU patients [19]. Additionally, therapy interruptions, individual pharmacokinetics, and machine-specific settings (e.g., dialysate flow, membrane properties) introduce variability not captured in the model. This explains the observed overestimation of clearance and further supports the necessity of therapeutic drug monitoring to guide individualized dosing in this complex population.

The safety of the dosing regimen employed (2 g q8h) in ADVOS and CVVHDF was supported by the absence of levels below therapeutic thresholds. However, a substantial proportion of trough levels exceeded 8 mg/L, raising concerns about potential toxicity. Neurotoxic and nephrotoxic effects have been reported at meropenem trough levels exceeding 44.45 mg/L and 64.2 mg/L, respectively (6), which was exceeded by one of our measurements in the ADVOS group. This high proportion of supra-therapeutic levels warrants further evaluation, especially in patients with limited residual renal function. As potential signs of neurotoxicity, delirium was documented in 10 out of 16 patients, and seizures occurred in two patients, both with underlying neurological disorders. It remains challenging to differentiate potential neurotoxicity from the consequences of critical illness. The patient with 62.8 mg/L meropenem trough level had severe pneumococcal pneumonia and acute renal failure and developed delirium but no convulsions.

The high variation of trough levels in the ADVOS group from 2.1 to 62.8 mg/L may be explained by several factors: the highly variable residual kidney function, the variable duration of treatment pauses of the ADVOS machine which might have caused accumulation of meropenem, different distribution volumes because of anasarca or capillary leak and dilution by a high fluid load are all possible explanations.

Generalizability is supported by the inclusion of a significant number of measurements (159), covering a range of clinical conditions, but caution is warranted in extrapolating the findings to antibiotics with different pharmacokinetic profiles, such as high protein binding or low intrinsic clearance agents (e.g. ceftriaxone, daptomycin).

This study has several limitations. First, the retrospective, single-center design limits its external validity. Second, the small sample size within subgroups, particularly for ADVOS therapy, reduces the power to detect statistically significant differences in meropenem clearance, especially in comparative analyses (e.g., t-tests). Third, the study did not account for interindividual variability in patient factors such as fluid status, inflammation, and other pharmacokinetic modifiers, which may influence meropenem levels. Together with the low number of patients, this may limit its generalizability and statistical interpretation. Two patients contributed data to more than one treatment group, this might introduce bias in between-group comparisons.

Moreover, the reliance on TDM performed under routine clinical conditions introduces potential inconsistencies in sample timing and handling. Since only values after dosing intervals without ADVOS interruptions were included, the total daily clearance may be overestimated since the daily pause to set up and exchange the ADVOS machine is not accounted for.

Future studies should investigate the pharmacokinetics of antibiotics with high protein binding and low intrinsic clearance during ADVOS therapy. Prospective, multicenter studies could provide more robust data and validate these findings across different ICUs. Moreover, detailed analyses of the impact of therapy interruptions and machine configurations on drug clearance are necessary to refine dosing strategies.

Pharmacokinetic modeling incorporating real-world TDM data could improve tools like *MeroEasy* to account for individual variations and therapy-specific factors. Additionally, studies comparing ADVOS with other extracorporeal modalities in critically ill patients with different organ dysfunction profiles (e.g., liver failure, intoxication) could help optimize dosing protocols for a broader range of clinical scenarios.

In conclusion, this study shows that patients with renal failure on ADVOS therapy at low blood flows of 100 mL/min receiving a high dose meropenem regimen (2 g q8h) have no risk of antibiotic underdosing and ADVOS therapy is safe for patients with severe infections. The study highlights the importance of TDM in extracorporeal therapies and critically ill patients, as the ideal dosing is unpredictable due to variable factors influencing drug levels. Our findings emphasize the need for personalized dosing regimens to balance therapeutic efficacy and safety and to impede overdosing.

## Supplementary Information

Below is the link to the electronic supplementary material.

Supplementary file1 (DOCX 37 KB)

## Data Availability

The data referred to during the study are available from the corresponding author on request.
