# Supplementary material for: Meropenem plasma concentrations in critically ill patients treated with the novel multi organ replacement therapy ADVOS
Source: Infection. 2025 May 21;53(5):2103–10. doi: 10.1007/s15010-025-02554-4 (PMC12460481; doi:10.1007/s15010-025-02554-4)
Supplement: Supplementary file 1 — Supplementary file1 (DOCX 37 KB) [file 15010_2025_2554_MOESM1_ESM.docx]

**Supplementary Information - MeroEasy Description**

Adapted from <https://doseeasy.de/>

MeroEasy is an educational software developed with the support of ADKA e.V. to aid in the training of physicians and pharmacists in understanding the impact of Meropenem dosing and pharmacokinetic formulas. MeroEasy uses pharmacokinetic population estimates, the Dettli method [1,2], and the Sawchuk-Zaske method [1] to calculate Meropenem doses. Doses can be calculated empirically (without prior concentration data, i.e. CL_estimated_) or using a single measured Meropenem concentration (i.e., CL_patient_.

MeroEasy determines pharmacokinetic parameters and dosing strategies for Meropenem through the following steps:

**Estimation of Clearance (Cl) and Volume of Distribution (Vd)**

Empirical dosing (no concentration): CL_estimated_

Meropenem clearance (CL) and volume of distribution (Vd) are estimated using published population data (Vd = 0.25 L/kg, CLpop = 13 L/h) [3]. Vd is calculated based on the ideal body weight plus 40% of excess weight. If actual body weight is lower than the ideal, actual body weight is used. Clearance is estimated based on renal function using the Cockcroft-Gault formula [1,4], applying ideal body weight plus 40% of excess weight, or actual body weight if it is below ideal (cut-off serum creatinine 0.6 mg/dL in women, 0.7 mg/dL in men to avoid overestimation in cachectic patients).

$${CL}_{creatinine, male}=\frac{140-age}{72\times{Creatinine}_{serum}}$$

$${CL}_{creatinine, female}=0.85\times{CL}_{creatinine, male}$$

Clearance is then calculated using the Dettli method based on individual renal excretion capacity Q (Q0 = 0.25) [2,3].

$$Q=Q_{0}+\frac{eGFR}{100 mL/min}\times(1-Q_{0})$$

$${CL}_{estimated}=Q\times{CL}_{POP}$$

Trough concentration available: CL_patient_

Vd is estimated as above. The measured concentration is matched to the infusion duration and corresponding half-life, and Clearance is calculated accordingly.

$$ke=-\frac{{ln(Meropenem}_{trough})-ln({Meropenem}_{min}+\frac{Dosis}{Vd})}{\Delta t}$$

$${CL}_{patient}=ke\times Vd$$

**References**

[1] Murphy JE. Clinical pharmacokinetics. 7th Ed. American Society of Health Pharmacists (ASHP), 2021.

[2] Dettli L. The kidney in pre-clinical and clinical pharmacokinetics. Jpn J Clin Pharmacol Ther 1984;15:241–54.

[3] Product information Meronem®, Germany. Updated May 2021.

[4] Cockcroft DW, Gault MH. Prediction of creatinine clearance from serum creatinine. Nephron 1976;16:31–41.
